# Development of an Integrated Chip for Automatic Tracking and Positioning Manipulation for Single Cell Lysis

**DOI:** 10.3390/s120302400

**Published:** 2012-02-23

**Authors:** Chao-Wang Young, Jia-Ling Hsieh, Chyung Ay

**Affiliations:** Department of Biomechatronic Engineering, National Chiayi University, No. 300, University Rd., East Dist., Chiayi 600, Taiwan; E-Mails: youngcw@mail.ncyu.edu.tw (C.-W.Y.); q_q11111@msn.com (J.-L.H.)

**Keywords:** electroosmotic flow, cell lysis, dielectrophoresis force, cell manipulation

## Abstract

This study adopted a microelectromechanical fabrication process to design a chip integrated with electroosmotic flow and dielectrophoresis force for single cell lysis. Human histiocytic lymphoma U937 cells were driven rapidly by electroosmotic flow and precisely moved to a specific area for cell lysis. By varying the frequency of AC power, 15 V AC at 1 MHz of frequency configuration achieved 100% cell lysing at the specific area. The integrated chip could successfully manipulate single cells to a specific position and lysis. The overall successful rate of cell tracking, positioning, and cell lysis is 80%. The average speed of cell driving was 17.74 μm/s. This technique will be developed for DNA extraction in biomolecular detection. It can simplify pre-treatment procedures for biotechnological analysis of samples.

## 1. Introduction

Cell membrane perforation and cell lysis are commonly used in cell engineering with applications in transgenic cells to obtain DNA. Cell lysis results in the destruction of cell membranes by mechanical, physical, chemical, and electrical methods. All currently used techniques caused sample damage or entail complex processes. Therefore, cell lysis directly occurring on a chip can simplify the pre-treatment procedures for biotechnological analysis of samples. In general, the researcher only selects the most special cell as sample, so we develop the integrated chip for automatic tracking and positioning manipulation of single cell lysis.

Obataya *et al.* used an ion beam etching technique to modify the probe tip diameter of the AFM to 200–300 nm [1]. The probe tip was used to carry out lysis of a living cell. The results showed that when a cell receives downward pressure from a probe tip at a distance of 1–2 μm, this is sufficient to complete cell lysis. Yoza *et al.* mixed *Escherichia coli* in a cell lysis solution by needle suction or heating to carry out cell lysis, as well as using bacterial magnetite to extract DNA from the micro-buffer liquid solution [2]. Youn *et al.* created compression and DNA storage areas in the microfluidic channel designed to cause cell deformation and rupture by extrusion [3].

The DEP force can capture and categorize cells under changing frequencies and intensities of the electric field. Then, the DEP force is generated to stretch or damage cells. Marszalek and Tsong used a high-frequency AC field to change the shape of a sea urchin egg, producing cell membrane vesicles and leading to cell division [4]. Lee and Tai designed their own cell lysis device that uses DEP force to lyse plant cells (20–40 μm) [5]. Kotnik used an electrical voltage method to conduct cell lysis. This technique involves the imposition of various electric fields to break up cell electroporation, leading to the rupture of cell membrane and the retrieval of the nucleus [6]. The main principle is based on the use of electric fields to compress cell membrane structures. Lapizco-Encinas *et al.* have shown that under appropriate electric field conditions, the capability to isolate live or dead colon bacillus into different regions is observed. Thus, their design can be used in sample concentration and aggregation [7].

The membrane dielectroporation technique involves the use of voltage control to cause reversible or irreversible electrical breakdown in cell membranes. This study will use membrane dielectroporation technique by DEP force to lyse cell.

## 2. Methods and Equipments

**Figure 1.**
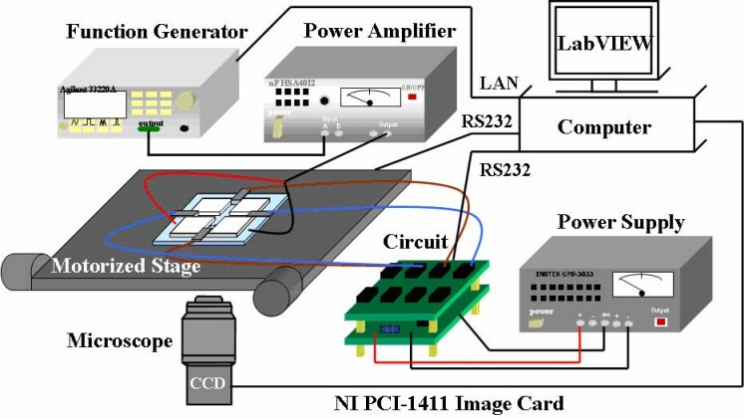
Diagram of the system configuration.

**Figure 2.**
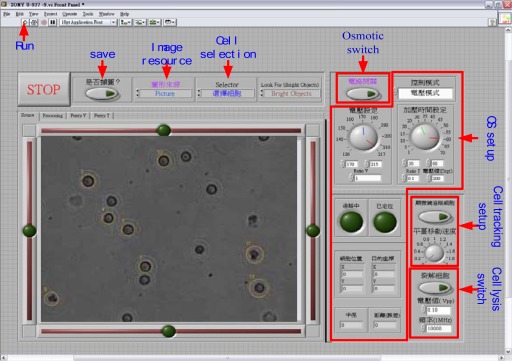
Panel of the man-machine interface.

The experiment was conducted on an inverse microscope. The electric platform, microscope CCD, and image acquirement card were used to capture real-time images. The local area network (LAN) control signal generator was used to project sine wave signals to lyse cells. The configuration of the entire system is shown in Figure 1. The man-machine interface package software in LabVIEW was used to create the machine vision control system to track a single cell and move it to a specific position and lyse the cells via electroporation. The man-machine interface panel is shown in Figure 2. The human-machine interface for the image processing of cell tracking is also shown in Figure 2, which illustrates an image as interpreted by the image processer as an image of a cell. Cells seen on the screen that have a yellow circle around their outer ring represent cells that have been interpreted successfully. If no yellow circle appears around the outer ring of a cell, then that cell has not been interpreted successfully by the system. First, the selected cells were tracked. The cells to be tracked were chosen from the capture screen using the mouse to mark the target by pointing and clicking on the selected cells. The tracking system locates the target cells to be tracked so as to complete the tracking behavior. The center of the target cell being tracked shows a cross symbol, and the tracking light in the LabVIEW program turns on to indicate that tracking is successful. The system automatically uses the target coordinates as the center, and uses the set value of the “Ratio” function on the control panel multiplied by the radius of the tracking cell as the localization radius, to draw a circle as the target criterion.

### 2.1. Image Processing

**Figure 3.**
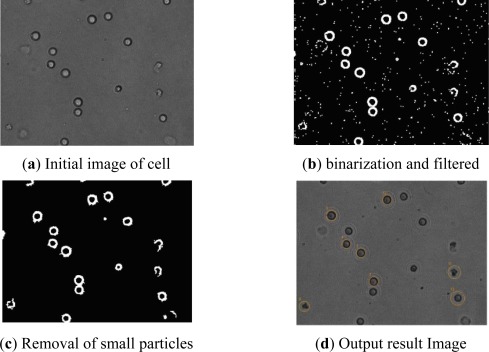
Result of image processing.

A NI PCI-1411 image card was used to acquire the images. Under a resolution of 640 × 480, 30 images could be acquired *per* second. In the beginning, the acquired image should be stored for observation, and the location and size of every cell should be found quickly by the program. Because the acquired image will have noise, the image should be filtered to eliminate the noise and obtain a smoother picture. In this experiment, the median filter was used, and binarization morphological processing of the image was conducted to realize accurate cell manipulation (as shown in Figure 3).

As for the procedure of tracking the cell, the target cell to be tracked is chosen. The system then compares the location coordinates of the mouse with respect to the location of all the circles to determine the target cell to be tracked and complete the tracking behavior. Then, the cell image is stored in the memory via a self-learn pattern function for the basis of identification, and the match pattern function is used to identify the cell in the next picture.

### 2.2. Electroosmotic Flow Control

When an electric field is applied to a liquid solution in a micro-channel because of the electric charge distribution in the electric double layer, the positively charged ions in the diffusion layer move toward the negative electrode under the electrical field influence of the Coulombic force. Furthermore, given the effect of viscous force, the movement of these ions drives the surrounding liquid so that the ions move together, causing fluid motion within the micro-channel. This is referred to as electroosmotic flow (EOF). The EOF rate of a liquid solution can be expressed as follows:

(1) $$\mu_{e0}=\frac{\varepsilon \zeta }{\eta }$$

*μ*_*e*0_ = solution EOF rate (m^2^·V^−1^·s^−1^)
*ε* = solution dielectric constant (kg·m·s^−2^·V^−2^)
*ζ* = zeta potential (V)
*η* = solution viscosity (viscosity, kg·m^−1^·s^−1^)

Taking into account these factors, we designed an EOF chip that can offer interface potential in conducting EOF. EOF was used to drive human histiocytic lymphoma (U937), quickly and precisely moving the cells toward the target lysis electrode. The automatic tracking of a single cell was achieved by controlling the strength and direction of the electric field using the system. After the cell was driven to the tip of the lysis zone, an appropriately controlled voltage was applied so that the cell can achieve irreversible electrical breakdown. The electric field generated by the AC in the tiny space induced the inner and outer cell membrane into generating opposing electrical distributions, thereby forming a squeeze pressure on the inner and outer cell membranes because of electrical attraction. This causes damage because of the thinning of the cell membrane, resulting in irreversible electrical breakdown from cell membrane rupture. The biochip used in this study adopts the micro-electromechanical systems process that evaporates the electrode onto a glass substrate. SU8-25 or film was used to produce a 10 mm long and 10 mm wide area. After the cell-containing micro-buffer solution was injected, the area was covered with a glass cover slip. Because EOF rate is proportional to the electric field, changing the electric field intensity was sufficient to control the EOF velocity. Control of the strength and polarity of the electric field was implemented to change the speed and direction of EOF that can drive cell movement in the micro-buffer solution, achieving movement of the designated target cell toward its destination.

**Figure 4.**
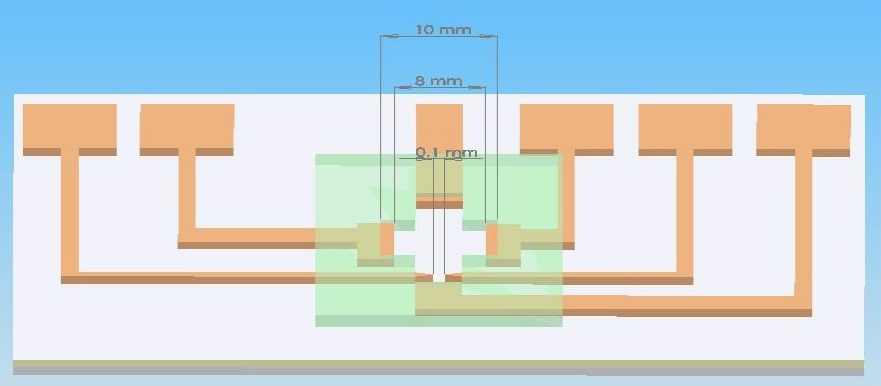
Diagram of an integrated chip.

The chip architecture diagram is shown in Figure 4. The electrode for cell lysis is a triangular palladium (Pd) electrode with a tip spacing of 100 μm. A program-controlled signal function generator produced the desired waveform, frequency, and voltage signals.

The purpose of electroosmotic control is to change the intensity and direction of the electric field to control the movement of the target cell in the solution. After the parameters of cell tracking were outputted from the systematic image part, the fuzzy logic method was used to achieve efficiency of cell movement.

**Figure 5.**
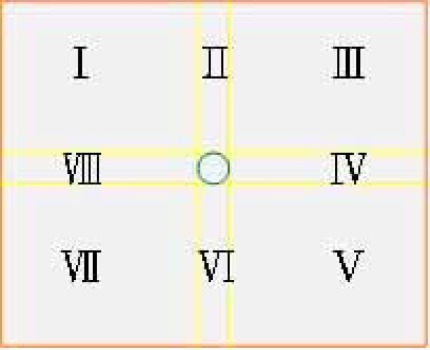
Partition of area.

As shown in Figure 5, the specific area was divided into eight parts by regarding the target range as the center. The direction of target cell movement is shown in accordance with the target area. I, III, V, VII represent the oblique voltage, and II, IV, VI, VIII represent the horizontal or vertical voltage. The direction of the electric field is dispersed from a high electric potential (positive electrode) to a low electric potential (negative electrode). In the fabrication of circuits, two output terminals with different voltages can be designed. If only the electric field with a single direction is applied, the electrode at another direction will be under a release state. As the tracked cell enters the target range, the system shows that the location has already been fixed and the operation of EOF is terminated. Thus, the tracked cell moves toward the lysis area gradually and is localized at the lysis area.

The AT89S51 chip was primarily used as the control circuit. RS-232 was used as the communication interface of the computer to receive and control two parameters. The first one is the movement direction, which uses the relay to change the direction of the electric field, and output it to the electrodes at four directions. The second is voltage information, which uses the digit to transfer analog signal changes to the output voltage.

We expect the movement of cells to be steady and fast. They should move fast when the distance is far from the localized point. They approach slowly when the distance is short. Because the movement rate of EOF is proportional to the electric field, the flow speed can be controlled by changing the intensity of the electric field. At the control rule, the fuzzy logic was used to calculate the optimal relationship between the distance and applied voltage [8,9].

**Figure 6.**
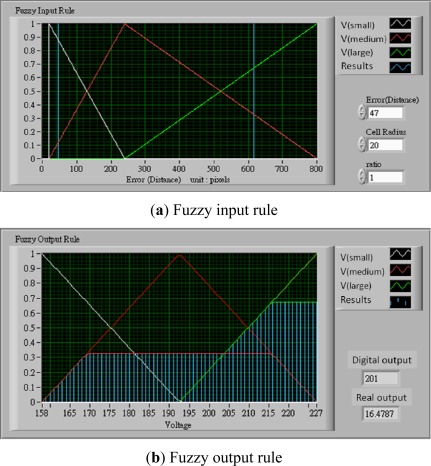
The fuzzy output rule in this control platform.

The voltage controller is developed basing on the center of gravity defuzzification. Given that the size of the acquired image frame is 640 × 480 pixels in length and width, its diagonal length is 800 pixels. The largest movement distance is 800 pixels when the tracked cell and target location are located at the end point of the diagonal, and the smallest movement distance is the radius of the tracked cell. The movement distance changes with cell size and radius ratio setting. The fuzzy membership functions are shown in Equation (2):

(2a) $$\mu_{\left(\mathrm{small}\right)}(x)=\frac{-(x-240)}{240-(\mathrm{Radius}\;x\;\mathrm{radio})},(\mathrm{Radius}\;x\;\mathrm{radio})\leq x\leq 240$$

(2b) $$\mu_{\left(\mathrm{medium}\right)}(x)=\frac{\left(x-\mathrm{Radius}\right)}{240-\mathrm{Radius}},\mathrm{Radius}\leq x\leq 240$$

(2c) $$\mu_{\left(\mathrm{medium}\right)}(x)=\frac{-(x-800)}{800-240},240\leq x\leq 800$$

(2d) $$\mu_{\left(\mathrm{large}\right)}(x)=\frac{\left(x-240\right)}{800-240},240\leq x\leq 800$$

where *Radius* is the radius of the tracked cell, and *ratio* is a scale parameter. We define, if the *Error (Distance)* value is less than the *Cell Radius* x *ratio,* it is successful for positioning. The value μ(*x*) is called the membership degree of *x* in the fuzzy set. The membership degree μ(*x*) quantifies the grade of membership of the element *x* to the fuzzy set. The value 0 indicates that *x* is not a member of the fuzzy set; the value 1 means that *x* is fully a member of the fuzzy set. The movement distance data were processed and transfer to output control voltage by the triangle center of gravity defuzzification [10,11]. The fuzzy output rule is shown in Figure 6. For example, if the voltage range is set as 9.2∼19.76 V (pixel range is 170∼215), when the movement distance between the tracked cell and the target location is 616 pixels (long distance), the radius is 20 and the ratio is 1 as shown in Figure 6(a). The value of μ_(medium)_(*x*) is 0.3286 and μ_(large)_(*x*) is 0.6714. The digital output (*D_o_*) is 201 according as the triangle center of gravity defuzzification. The output voltage (*V_o_*) is relayed to the *D_o_*, as is shown in Equation (3):

(3) $$V_{0}=0.2357\;D_{o}-30.897$$

where *D_o_* = distance (pixels), *V_o_* = output voltage (voltage). The real *V_o_* is 16.48 volts, as shown in Figure 6(b).

### 2.3. Cell Lysis

The cell samples used in this study were human histiocytic lymphoma U-937. The cell culturing medium contained the necessary nutrients, appropriate pH, and osmotic pressure at 37 °C and 5% CO_2_. Pretreated cell solution was injected to the cell injection end located at the left hand side of chip. Then, 100 μL of 0.2% glucose solution was used to fill the cross-channel. Subsequently, program began identifying the cell closest to the intersection of the cross-channel. The switch of the EOF was turned on to control the movement of the target cell.

**Figure 7.**
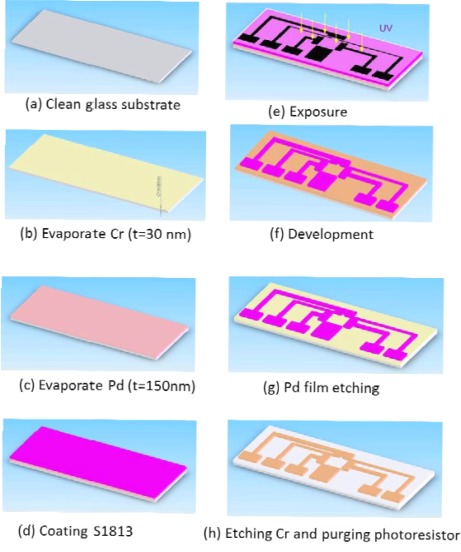
Fabrication of Pd electrode.

We used Pd electrodes to construct an EOF-driven microelectrode. A layer of chromium film should first be evaporated onto a glass substrate before the evaporation of the Pd film. The spacing between the triangle tip electrodes was maintained at 100 μm (width), as shown in the mask map in Figure 7. The power amplifier generated a sine wave signal (AC) with a voltage of 15 V and a frequency of 1 MHz. The electric field generated by the AC in the tiny space induced the inner and outer cell membrane into generating opposing electrical distributions, which forms a squeeze pressure on the inner and outer cell membranes because of electrical attraction. This causes damage given the thinning of the cell membrane, resulting in irreversible electrical breakdown from cell membrane rupture. Suitable voltage control can be used to control the damage outside or inside the cell. The principle of cell lysis by voltage lies in compressing the cell membrane structure primarily with the mechanism of the electric field. When the electric field is large enough, the opposite electricity is imposed upon the interior and exterior membranes of the cell. Then, the interior and exterior membranes become thinner because of the compressed pressure. After this, the cell membrane is destroyed [5].

**Figure 8.**
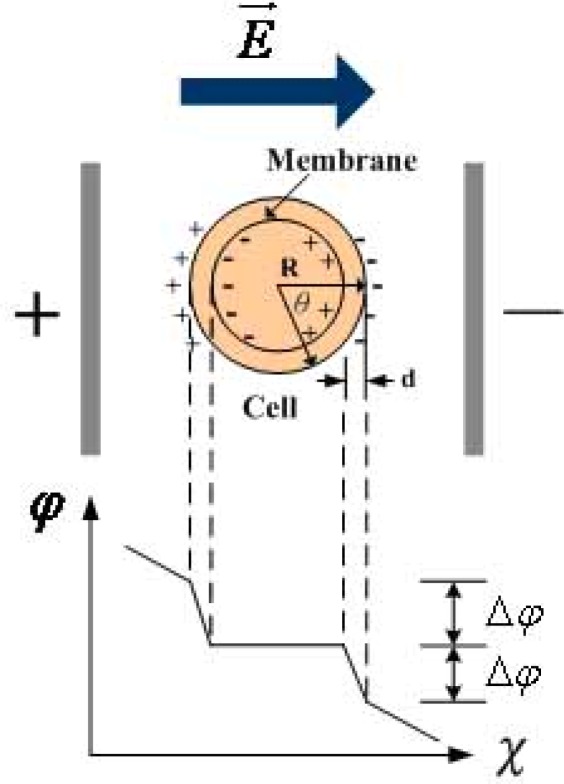
Electroporation potential of external electric field.

Figure 8 shows the electroporation potential induced by the applied electric field. The electroporation potential is closely related to the applied electric field, as is shown in Equation (4):

(4) Δϕ=−1.5 E R cos θ

where: *E*: applied electric field (V/m);

*R*: cell radius (m);
*θ*: angle between the direction of the electric field and normal line direction of the cell membrane.

### 2.4. Control of Tracking, Positioning, and Cell Lysis in an Integrated Chip

The micro-buffer solution is a glucose solution with DI water with a weight percentage concentration of 2%. The cell solution removed from the incubator must be pre-processed before it can be mixed with the blended micro-buffer solution. The mixed cell solution was dropped onto a clean glass substrate, which was set aside for continuous observation for 15 min for cell survival status. The steps were as follows:

1. The Petri dish covered with cells were removed after a week of culturing.
2. The cell solution (200 μL) was retrieved from the Petri dish and placed it in to a 1.5 mL micro centrifuge tube.
3. The centrifuge machine was set to 1,800 rpm × 6 min to centrifuge the cell solution.
4. When centrifugation was completed, the supernatant was extracted from the cell solution, and the cells were deposited onto the bottom alone.
5. A completely mixed micro-buffer solution (200 μL) was added and mixed evenly.

**Figure 9.**
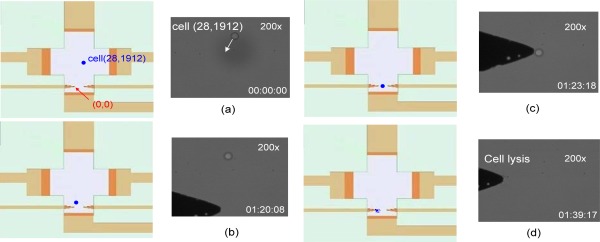
Cell driving, positioning and lysis process.

The experimental procedure was as follows. First, the above-mentioned system was constructed, after which the chip was fixed onto the X-Y platform. The connection between EOF and the self-made electrical circuit was controlled. The tip of the metallic electrode was attached to the power amplifier. Then, the following steps were performed:

1. Cell injection: 2 μL of pre-processed cell solution was injected into the on-chip cell driving region.
2. Injection of the micro-buffer solution: Glucose was taken as the working solution with a weight percentage of 2% and injected into the cell micro-buffer solution work area.
3. Addition of glass cover slip: A glass cover slip was placed on the working solution area. This allows the working solution to smoothly cover the entire region in order to prevent the loss of the effectiveness of EOF due to the inability of the micro-buffer solution to be fully exposed to the working electrode.
4. Electrically-driven EOF: The targeted cells locked in by the program were accessed. Then, the EOF switch in the program that controls the EOF flow was turned on in order to move the target cells toward the destination (cell lysis at the tip of the electrode). When the cells were driven to a position before the tip of electrode, the EOF switch in the program was turned off in order to stop the EOF from driving the cells further.
5. Cell lysis: The program was used to set the cell lysis parameters (voltage signal waveform, voltage magnitude, and frequency). The select and write functions of the program used LAN to send the set parameters to the signal function generator in order to produce a voltage signal. The cell lysis process was observed as displayed on the computer screen and recorded as the experimental video.
6. Upon cell lysis completion, the program was terminated. Figure 8 shows cell lysis after the cells have been moved to the cell lysis area. Figure 9(a) sets the coordinates of the left cell lysis electrode tip to (0, 0), where the coordinates of the cell relative to its original position are (28, 1,912). The driving direction is shown in the figure. Figure 9(c) shows the designated drive-to location for the cell. Figure 9(d) shows a successful cell lysis occurring right in front of the designated electrode.

## 3. Results and Discussion

Cell membranes possess capacitance characteristics. When the frequency of the electrical current passing through a cell is relatively low, the cell membrane will be of high-impedance type, indicating that the current loses the ability to pass through the cell. Conversely, a high-frequency current will pass through the cell membrane unimpeded. In this experiment, a tip electrode made of Pd was connected to an electrical current of 15 V at frequencies of 1 Hz, 100 Hz, 1 kHz, 10 kHz, 50 kHz, 100 kHz, 200 kHz, 400 kHz and 1 MHz for cell lysis. Finally, at a frequency of 1 Hz, bubbles were produced so this frequency was excluded. When the frequency was 50 kHz, the cells began to immediately bounce after electrode adsorption. Cell lysis took place during this adsorption process. When the frequency was higher than 100 kHz, almost no cell bounce was observed after attraction by the tip electrode. However, at a frequency of 200 kHz, cell bounce phenomenon re-occurred. When the frequency was set to above 400 kHz, cells began to lyse after direct adsorption, with almost no bounce phenomenon observed. Thus, it can be posited that at frequencies higher than 400 kHz, the reliability of cell lysis directly after electrode adsorption is relatively higher.

**Figure 10.**
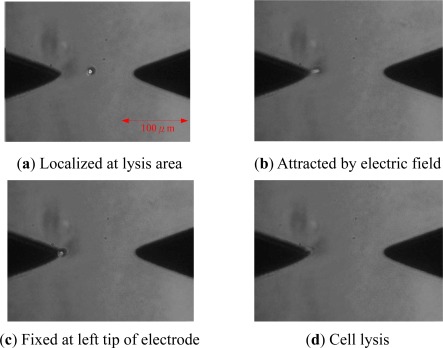
Cell lysis process.

Although the cell was localized in the middle of the electrodes, the fabrication of the microelectrode was unable to achieve the perfect condition. Therefore, it cannot produce a uniform electric field and causes the electrophoresis phenomenon. Thus, the cell will be attracted by the largest area of the electric field. As shown in Figure 10, the cell will move to the left or right randomly for cell lysis.

The experimental results show the experimental statistics of time required for tracking and positioning of the targeted cells to the cell lysis area and the success rate of localization under 1 MHz and 15 V. This study designated the tip electrode for cell lysis at the origin, with coordinates set to (0, 0). A tracking cell was randomly selected. A computer program was used to obtain the center coordinates of the tracking cell. With this data, the distance between the cell and the designated tip electrode can be calculated. We performed 30 separate experiments. If the randomly selected tracking cells could not be successfully moved, then the operation was considered a failure. If the tracking cells became stuck during the moving process, it was also considered a failure. Within the 30 experiments, there were 24 successes and six failures. The overall successful rate of cell tracking, positioning, and lysis was 80%. The average driving speed was 17.74 μm/s, as shown in Table 1.

There were 24 instances of successful driving of the tracking cells. In these 24 equal cell lysis experiments, it was found that if the cell lysis parameters set to the electric field of 1.5 × 10^5^ V/m (electric field conversion calculated using Equation (3), the distance between the two electrodes is 100 μm, so the electric field is 1.5 × 10^5^ V/m) and frequency equals 1 MHz, cell lysis was 100% successful in a location in front of the designated electrode.

**Figure 11.**
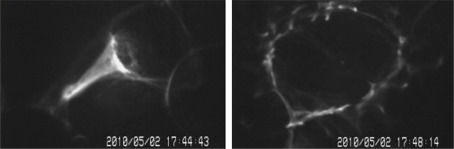
Filament DNA image.

After cell lysis, the fluorescent dye YO-PRO-1 was added to the lysis absorbing cell solution, which was then observed under a microscope. Through a 100× immersion lens and B-cell nucleus radiation, the DNA image of the post-lysis U937 cell could be observed in Figure 11. This proves that the trans-membrane formed by the combination of the tip microelectrode and AC in this system architecture can succeed in cell lysis for intracellular DNA extraction.

## 4. Conclusions

This study used NI LabVIEW software for single cell tracking, positioning, and monitoring in combination with circuit design with EOF control to accurately position cells to the lysis area at the tip of electrodes, creating an appropriate trans-membrane potential and successfully achieving cell lysis. Using experimentation, the optimal cell lysis was achieved using a voltage of 15 V and a frequency of 1 MHz. The cell driving success rate in this experimental architecture was 80%, with an average driving speed of 17.74 μm/s.

## Figures and Tables

**Table 1.** The time and speed of cell lysis.

No.	**Distance (μm)**	**Time (s)**	**Speed (μm/s)**	**Success**
1	286.14	15.19	18.83	o
2	0	0	0	x
3	188.36	6.1	30.87	o
4	1,176.29	75.02	15.67	o
5	215.23	26.12	8.24	o
6	1,912.20	83	23.03	o
7	897.55	60.11	14.93	o
8	235.03	11.2	20.98	o
9	0	0	0	x
10	448.64	17.04	26.32	o
11	1,083.49	82.08	13.20	o
12	892.32	48.23	18.50	o
13	976.38	93	10.49	o
14	0	0	0	x
15	1,643.63	87.22	18.84	o
16	144.40	29.28	4.93	o
17	764.32	37.22	20.54	o
18	816.97	28.1	29.07	o
19	505.15	45.23	11.17	o
20	0	0	0	x
21	283.67	19.18	14.79	o
22	2,791.78	93.1	29.99	o
23	1,491.36	62.19	23.98	o
24	174.14	42.29	4.12	o
25	0	0	0	x
26	0	0	0	x
27	200.68	29.12	6.89	o
28	848.07	32.18	26.35	o
29	148.63	23.2	6.41	o
30	713.90	44.18	16.16	o
	Successful rate: 80.00%		Average speed: 17.74 μm/s	
